# Integrating Telemedical Supervision, Responder Apps, and Data-Driven Triage: The RuralRescue Model of Personalized Emergency Care

**DOI:** 10.3390/jpm15070314

**Published:** 2025-07-14

**Authors:** Klaus Hahnenkamp, Steffen Flessa, Timm Laslo, Joachim Paul Hasebrook

**Affiliations:** 1 Clinic for Anaesthesia, Intensive Care, Emergency and Pain Medicine, University Medicine Greifswald, 17475 Greifswald, Germany; klaus.hahnenkamp@med.uni-greifswald.de; 2 University Greifswald, 17489 Greifswald, Germany; 3 University Greifswald, District Vorpommern-Greifswald, 17489 Greifswald, Germany; timm.laslo@kreis-vg.de; 4 zeb business school, University Witten-Herdecke, 48153 Münster, Germany; jhasebrook@zeb-bs.de

**Keywords:** emergency care, personalized medicine, tele-emergency physician, rural health, responder app, digital triage, EMS systems, context-aware care, implementation science, system integration

## Abstract

**Background/Objectives**: This study aimed to evaluate a regional implementation project for rural emergency care (RuralRescue) and to examine how its components and outcomes may support personalized approaches in emergency medicine. While not originally designed as a personalized medicine intervention, the project combined digital, educational, and organizational innovations that enable patient-specific adaptation of care processes. **Methods**: Conducted in the rural district of Vorpommern-Greifswald (Mecklenburg–Western Pomerania, Germany), the intervention included (1) standardized cardiopulmonary resuscitation (CPR) training for laypersons, (2) a geolocation-based first responder app for medically trained volunteers, and (3) integration of a tele-emergency physician (TEP) system with prehospital emergency medical services (EMSs). A multi-perspective pre–post evaluation covered medical, economic, and organizational dimensions. Primary and secondary outcomes included bystander CPR rates, responder arrival times, telemedical triage decisions, diagnostic concordance, hospital transport avoidance, economic simulations, workload, and technology acceptance. **Results**: Over 12,600 citizens were trained in CPR and the responder app supported early intervention in hundreds of cases. TEPs remotely assisted 3611 emergency calls, including delegated medication in 17.8% and hospital transport avoidance in 24.3% of cases. Return of spontaneous circulation (ROSC) after out-of-hospital cardiac arrest (OHCA) was achieved in 35.6% of cases with early CPR. Diagnostic concordance reached 84.9%, and documentation completeness 92%. Centralized coordination of TEP units reduced implementation costs by over 90%. Psychological evaluation indicated variable digital acceptance by role and experience. **Conclusions**: RuralRescue demonstrates that digitally supported, context-aware, and regionally integrated emergency care models can contribute significantly to personalized emergency medicine and can be cost-effective. The project highlights how intervention intensity, responder deployment, and treatment decisions can be tailored to patient needs, professional capacity, and regional structures—even in resource-limited rural areas.

## 1. Introduction

The provision of timely, high-quality emergency medical services (EMSs) remains a major challenge in rural areas of Europe, where structural limitations such as long distances, staff shortages, and aging populations place disproportionate burdens on healthcare infrastructure. A recent systematic review found that rural populations in high-income countries experience significantly longer EMS response times and lower survival rates in time-critical emergencies such as cardiac arrest or stroke compared to urban populations [1].

In Germany, the district of Vorpommern-Greifswald, located in the northeastern state of Mecklenburg-Western Pomerania (Mecklenburg-Vorpommern), exemplifies this situation. With a land area of approximately 4000 square kilometers (1544 square miles) and a population density of just 60 inhabitants per square kilometer (155 inhabitants per square mile), it represents a structurally underserved rural region [2]. EMS provision in such regions is constrained by both geographical barriers and a shrinking pool of emergency physicians who are trained to deliver advanced prehospital interventions.

The German EMS follows a two-tier system: paramedics (with 3 years of training) attend all emergencies, and emergency physicians are dispatched separately under the “rendezvous system” when required for advanced interventions [3]. In practice, however, data show that physicians are dispatched in 46 percent of all EMS cases, but in up to one third of these deployments, no physician-specific intervention is ultimately needed [4]. This mismatch leads to inefficient resource use, delays in response, and regional disparities in care availability, particularly in rural areas [5].

In the district of Vorpommern-Greifswald, these problems are especially pronounced. According to project data, more than 60 percent of the district’s territory lies beyond the 10 min emergency physician arrival time recommended in national guidelines, and long gaps in 24/7 physician coverage have been documented in several remote sub-regions [2,6]. These care deficits are not limited to this district: the official transfer recommendation by the Federal Joint Committee (G-BA) identifies similar challenges across multiple rural regions in Germany, including insufficient physician coverage, prolonged EMS response times, and gaps in advanced care provision [7].

To address these structural shortcomings, the RuralRescue (Land|Rettung) initiative piloted a comprehensive model for telemedicine-supported EMSs in Vorpommern-Greifswald, funded by the Innovation Fund of the Federal Joint Committee (Gemeinsamer Bundesausschuss, G-BA). At its core is the tele-emergency physician (TEP) system, which enables paramedics on site to receive real-time medical supervision via video, audio, and diagnostic data transmission. Between 2018 and 2020, over 9000 emergency cases were supported by tele-emergency physicians. In 24% of these, patient transport to hospital could be avoided, and in 18 percent, pharmacological interventions were delegated remotely [2,6].

Beyond remote supervision, the RuralRescue project integrates a smartphone-based first responder system, structured triage support, and role-specific training for EMS personnel, creating a flexible, data-driven model that aligns with the principles of personalized emergency medicine. Personalized emergency care is understood here as the dynamic adaptation of triage, treatment intensity, and care location to the real-time clinical, geographic, and contextual characteristics of each case [8,9].

This article introduces the RuralRescue project as a real-world implementation of personalized emergency medicine in rural environments. Drawing on data from over 250,000 EMS operations, it examines how telemedical innovation can improve equity, efficiency, and individualization in acute care.

### 1.1. Digital and Telemedical Solutions in Rural EMS: Evidence and Integration

Inequities in emergency medical care between urban and rural regions have been extensively documented in recent years. Multiple studies have shown that rural populations experience longer ambulance response times, limited access to advanced prehospital interventions, and lower survival rates in emergencies such as out-of-hospital cardiac arrest or stroke, compared to urban areas [10,11]. These disparities are particularly pronounced in geographically large and sparsely populated districts, where access to on-site emergency physicians is limited due to staff shortages and travel distances [10,12]. To address these systemic challenges, several regions have begun to implement telemedicine-based solutions in emergency services. These include tele-emergency physician systems, smartphone-based first responder alert applications, and digitally supported triage protocols. All of these have shown the potential to improve operational efficiency while enabling individualized medical decision-making at the point of care [12,13,14].

In the German context, tele-emergency physician systems have been piloted and evaluated in regions such as Aachen, Gütersloh, and others. These models enable emergency medical technicians and paramedics to connect remotely with a physician during an emergency. Vital parameters such as electrocardiogram (ECG), blood pressure, oxygen saturation, and high-definition video are transmitted in real time. Based on this data, the physician provides clinical decision support, authorizes pharmacological treatment, and may guide the selection of a destination hospital. In controlled evaluations, these systems have been associated with reduced unnecessary dispatches of on-site physicians, improved triage precision, and shorter delays to evidence-based interventions, particularly in acute myocardial infarction and polytrauma scenarios [15,16,17]. Furthermore, observational studies show high satisfaction and acceptance among paramedics, physicians, and patients, and demonstrate that delegated interventions such as analgesia or antihypertensive treatment can be delivered safely in the field under remote supervision [13].

Complementary to physician support systems, mobile first responder applications have been developed to engage trained laypersons and off-duty medical professionals in early resuscitation. Such systems rely on GPS-based activation of responders who are in close proximity to the emergency. These responders are alerted to suspected cardiac arrests or other life-threatening events, allowing them to initiate cardiopulmonary resuscitation (CPR) before the arrival of formal EMS teams. Studies report improved rates of bystander CPR and significant reductions in no-flow time for patients in rural areas where EMS response is delayed [18,19]. These systems represent a form of spatially and temporally adaptive response, in which the mobilization of human resources is tailored to each emergency’s geographic and temporal context [12].

**Figure 1:**
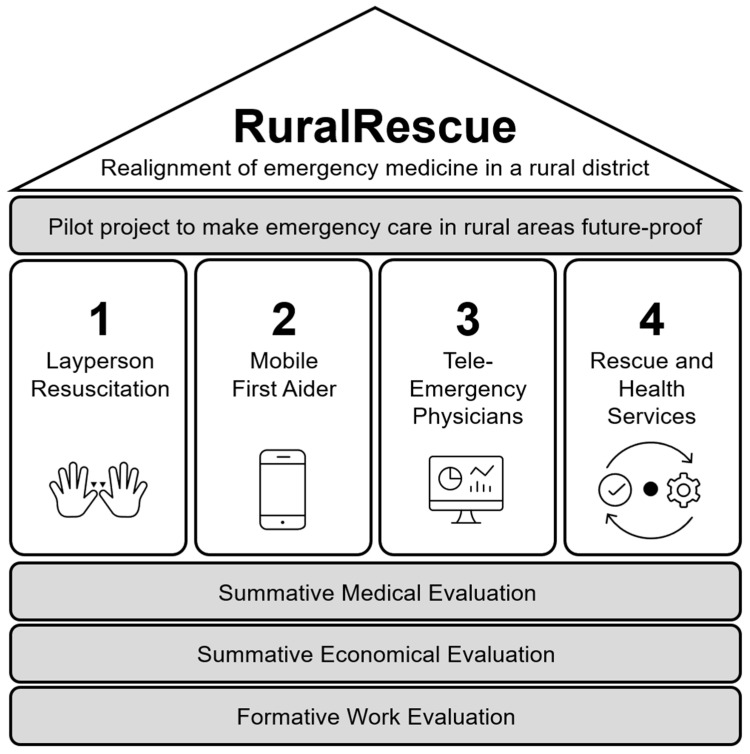
Structural framework of the RuralRescue (Land|Rettung) project.

The RuralRescue project, implemented in the rural district of Vorpommern-Greifswald, represents the first fully integrated operational model in Germany that combines these technologies into a coherent system architecture (see Figure 1). Within the project, community-based first aid training, geolocation-based responder activation, real-time tele-emergency physician services, and tailored education for EMS professionals were systematically linked. This integration was not only technical but also procedural and organizational, allowing the participating actors to coordinate their actions across the emergency care chain. According to project evaluation data, over 9000 emergency cases were supported by the tele-emergency physician service between 2018 and 2020, with transport to hospital avoided in approximately 24 percent of cases, and delegated medication administered in 18 percent [6,12].

This combination of technologies and processes enables personalization of care in several respects. The involvement of the tele-emergency physician allows clinical decisions to be made based on real-time patient data, rather than standardized protocols alone. The mobile responder system adapts the alerting process to the location and qualifications of available individuals. Digital triage tools and structured communication protocols facilitate case-specific treatment escalation or de-escalation. The role-specific training programs for paramedics and dispatchers enhance collaborative competence and ensure that delegation and supervision are matched to individual team configurations and experience levels [20].

While each of these technologies has been deployed separately in earlier projects, the RuralRescue project is the first to evaluate their integration under real-world conditions in a structurally underserved rural area. As such, it provides both empirical insight and a transferable model for the implementation of personalized emergency medicine across the full span of the prehospital care pathway.

### 1.2. Aim of the RuralRescue Project

The RuralRescue project was implemented in the rural district of Vorpommern-Greifswald between 2016 and 2020 under the Innovation Fund of the German Federal Joint Committee. The Innovation Fund was established to support implementation research for novel forms of care that go beyond existing standard provision. Its primary objective is to enable the transfer of evidence-based medical strategies into the statutory health insurance system, particularly in structurally underserved regions. In this context, RuralRescue aimed to redesign emergency medical services in rural areas through a coordinated four-pillar model: (1) public training in cardiopulmonary resuscitation (CPR), (2) smartphone-based activation of qualified first responders, (3) deployment of a tele-emergency physician (TEP) system, and (4) structural integration of emergency services with out-of-hours primary care [21].

The central aim of the project was to safeguard high-quality emergency medical care in rural areas by reducing the “therapy-free interval”—the time between the onset of an emergency and the beginning of effective intervention—while maintaining statutory response time thresholds [21]. Over 12,600 residents of the region were trained in CPR techniques, the RuralRescue mobile application was activated in hundreds of alerts, and more than 3600 emergency cases were supported remotely by a tele-emergency physician during the implementation phase [21]. These interventions were evaluated for their medical effectiveness, organizational feasibility, and potential for transferability to other rural districts [21].

Although the project was not originally conceptualized within the paradigm of personalized medicine, many of its key mechanisms are highly consistent with this approach. Real-time telemedical triage based on transmitted patient data allows for clinical decisions tailored to individual physiological profiles. The first responder system adapts emergency notifications based on geographic proximity and responder qualifications. Medication delegation, when conducted under remote supervision, is adjusted to the competencies of the on-scene personnel and the clinical needs of the patient. In the context of this study, personalized emergency medicine refers to the alignment of emergency medical interventions with individual patient needs, situational context, and regional infrastructure. This includes real-time tailoring of diagnostics, triage, and therapy based on clinical data, geographic conditions, and professional competencies, supported by digital tools and system-level adaptations.

This article evaluates the RuralRescue project as a model of digitally supported emergency medicine in a rural context and examines its alignment with the principles of personalized care. In doing so, it contributes to ongoing discussions about how data-driven, adaptive emergency services can improve individual outcomes while addressing systemic access gaps in structurally disadvantaged regions.

## 2. Materials and Methods

### 2.1. Intervention Design and Personalization Mechanisms

The RuralRescue project was implemented in the rural district of Vorpommern-Greifswald in northeastern Germany. It aimed to improve access to timely and high-quality emergency care in structurally underserved areas by shortening the “therapy-free interval”—the period between the onset of a medical emergency and the delivery of effective therapeutic intervention [22]. The intervention was based on a four-pillar model, of which three pillars were fully implemented and evaluated.

Pillar 1: Community-based layperson training in cardiopulmonary resuscitation (CPR). This component sought to increase first-aid competence in the general population by offering training courses in basic life support and the use of automated external defibrillators (AEDs). Over 12,600 residents participated in structured CPR training sessions, which were tailored to different age groups and learning settings [22]. This component indirectly contributed to personalized emergency response by expanding the pool of trained bystanders available for geolocation-based alerts (see Pillar 2), while also addressing community-specific preparedness levels.

Pillar 2: Smartphone-based first responder activation system. A mobile application called “Land|Retter” (German for “Rural Saver”) was developed and deployed to alert nearby trained volunteers or off-duty healthcare professionals to time-critical emergencies, particularly out-of-hospital cardiac arrest (OHCA). The system used real-time GPS data to identify and notify individuals within a defined radius of the incident. In its later development stages, the alerting logic also considered responder qualifications (e.g., layperson, paramedic, physician), allowing contextual adaptation of mobilization strategies [22,23]. This geospatial and role-based personalization of emergency response enabled earlier intervention based on both the physical proximity and the skill profile of responders [23].

**Figure 2:**
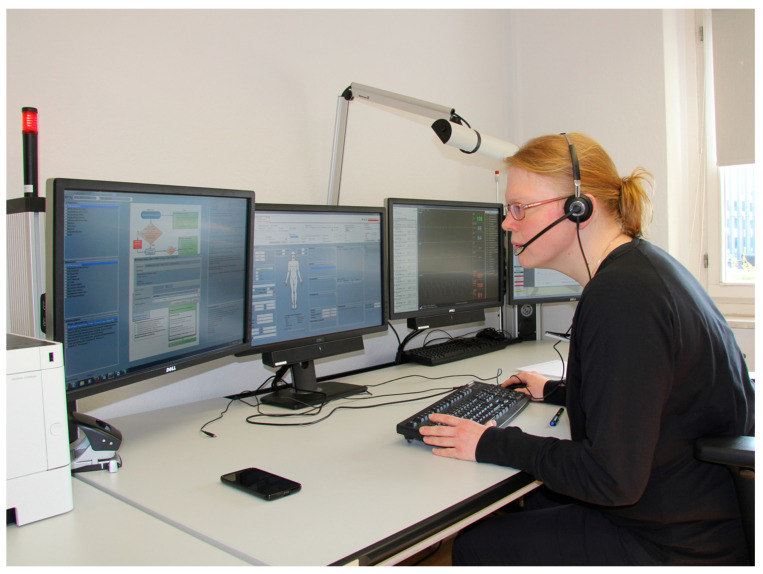
Working station of a tele-emergency physician (TEP; photo taken from [15]).

Pillar 3: Tele-emergency physician (TEP) system. To supplement and optimize the use of on-scene emergency physicians, a tele-emergency physician service was introduced. Paramedics were equipped with secure digital devices that enabled real-time transmission of clinical data—including ECGs, vital signs, and patient assessments—to a remotely located physician. Using a dedicated documentation interface (TeleDoc; see Figure 2), the tele-emergency physician could supervise diagnostics, delegate medication, and guide hospital triage decisions in accordance with standardized but situation-sensitive protocols [22,24]. This component allowed for clinical personalization of emergency care, with therapeutic decisions based on the real-time physiological and contextual information of the patient. Medication delegation was adapted to both the clinical profile of the patient and the training level of the on-scene personnel [24].

Pillar 4: Integration with out-of-hours primary care. The project’s fourth conceptual pillar, closer coordination between EMS and on-call primary care physicians, was discussed with experts for regional adaptation as a part of the formative evaluation [22].

### 2.2. Evaluation Design

The evaluation of the RuralRescue project followed a multi-perspective, implementation science approach to assess the effectiveness, efficiency, and work–system integration of a digitally supported emergency medical model in a rural context. The RuralRescue evaluation employed a pre–post design without a control group, which is a widely used and accepted strategy in implementation research under real-world conditions where randomized allocation is neither feasible nor ethically appropriate. As emphasized in prior studies, such designs are particularly valuable for evaluating system-level innovations in complex, context-dependent settings, where the primary objective is to assess change under routine operational conditions rather than to isolate causal effects under controlled conditions [25,26]. Given the structural and technological complexity of the intervention, the evaluation was organized into three complementary domains: (1) a summative medical evaluation of clinical and operational outcomes, (2) a summative economic evaluation of resource use and cost-efficiency, and (3) a formative work psychological and organizational evaluation focused on digital acceptance, knowledge transfer, and collaborative practice adaptation.

#### 2.2.1. Summative Medical Evaluation

The medical evaluation aimed to determine the clinical performance of the RuralRescue intervention using predefined tracer diagnoses and care pathway indicators. Key outcomes included the therapy-free interval (time from emergency onset to first therapeutic intervention), arrival times of EMS units and first responders, and effectiveness of TEP interventions, such as delegated medication and transport decisions [23]. For out-of-hospital cardiac arrest (OHCA) cases, return of spontaneous circulation (ROSC) served as a critical clinical endpoint [23].

Secondary indicators encompassed the rate of hospital admission versus non-transport, diagnostic accuracy in prehospital triage, and completeness and quality of digital documentation, including use of the dedicated TeleDoc system for physician–paramedic interaction logging. The evaluation used a matched-pairs design and time-series analysis, comparing TEP-supported cases to standard EMS contacts, and current performance to historical data from 2014 to 2017 [23].

Inclusion criteria encompassed all EMS operations in the district of Vorpommern-Greifswald between January 2018 and December 2021 where at least one RuralRescue component (CPR training, Land|Retter alerting, or TEP support) was activated and full clinical documentation was available. Exclusion criteria included pediatric emergencies (<6 years), trauma-related incidents, and cases with incomplete datasets. A total of 3611 TEP-supported cases and over 9000 app activations were analyzed, representing >85% of eligible cases after exclusion filtering. Recruiting followed a census-based approach using retrospective extraction from the central EMS IT system. Patient follow-up surveys and structured interviews were conducted using stratified convenience sampling with >600 completed cases. A post hoc power analysis for primary endpoints (e.g., ROSC, pain reduction) indicated power >0.80 at α = 0.05 for medium effect sizes (Cohen’s d ≥ 0.4).

#### 2.2.2. Summative Economic Evaluation

The economic evaluation of the RuralRescue model focused on the cost structure, utilization, and scalability of the TEP system in rural emergency medical services. Using operational data from Vorpommern-Greifswald and simulation modeling, this study assessed whether digitally supported, on-demand physician care could be delivered both effectively and efficiently under real-world conditions.

The economic evaluation examined the cost-effectiveness and systemic resource impact of the RuralRescue model compared to standard emergency care. Areas of focus included the implementation and operational costs of the TEP infrastructure, mobile responder alert system, and training programs, as well as savings from avoided on-scene physician deployments and unnecessary hospital admissions [24].

Economic modeling used full-cost accounting and queuing theory to simulate different TEP deployment strategies (centralized vs. decentralized) and assess effects on availability, capacity, and budgetary requirements. Primary data sources included complete deployment logs, billing data, and resource consumption reports from the emergency services IT platform. Stakeholder input was collected through ten semi-structured interviews with dispatch supervisors, EMS leads, and administrative staff. Cost structures were modeled based on full-cost accounting, differentiating between fixed and variable components (e.g., infrastructure, staff, licensing). The queue modeling (M/M/k structure with ∞, FIFO) simulated TED coverage scenarios under varying regional configurations. The analytical dataset covered all 3611 TEP interventions and was complemented by survey data on standby resource utilization and case complexity. Although no prospective recruitment occurred, the full case census provided sufficient data granularity to simulate system behavior across scalability tiers. Model fit was validated through sensitivity analyses. Test power was not relevant due to the deterministic nature of simulation outputs.

#### 2.2.3. Formative Work Psychological and Organizational Evaluation

The formative evaluation addressed the subjective and behavioral adaptation of key stakeholders—paramedics, dispatchers, emergency physicians, and trained lay responders—to the digitally transformed care model. It assessed technology acceptance, perceived workload, knowledge transfer, and regional acceptance of the intervention.

The formative evaluation applied a triangulated mixed-methods design combining psychometric self-assessments, role-specific surveys, and expert workshops at two timepoints (pre- and post-implementation) [19]. Recruitment was purposive and role-stratified, targeting all 292 EMS professionals employed in the district during the project period, including paramedics, dispatchers, physicians, and administrative staff. The final sample included 164 fully completed surveys (response rate ≈ 56%), ensuring representativeness across roles and experience levels. The instruments included the NASA-TLX (for workload), TAM2 (for technology acceptance), and validated Likert-based scales for job satisfaction and knowledge transfer behavior. Test power was >0.80 for detecting small-to-medium effects (Cohen’s f ≥ 0.20, α = 0.05) in repeated measures and interaction effects. Five moderated expert discussions involving 38 stakeholders applied the GEM Assay to analyze regional structural alignment and system readiness. Qualitative data were evaluated using thematic coding and triangulated with survey findings to identify barriers and enablers of system adaption.

Technology acceptance was measured focusing on perceived usefulness and ease of use of TEP systems and the Land|Retter application. Workload and job satisfaction were evaluated using the NASA Task Load Index (NASA-TLX), investigating the interrelationship of workload levels with willingness to adopt digital tools [27].

Knowledge transfer and the use of knowledge sources were examined using a framework based on the KODE^®^ Competence Atlas, which distinguishes between explicit and implicit, as well as personal and organizational, knowledge domains. This approach was applied to better understand how work satisfaction influences both formal and informal knowledge sharing across professional boundaries [19].

Regional acceptance and structural compatibility were assessed using the GEM Assay, which evaluates system alignment across three domains: groundings (e.g., emergency infrastructure), enterprises and parties involved in EMS delivery, and the specific characteristics of the target region [23]. This method was applied to examine how well the RuralRescue model fits the local operational context and to assess its acceptance across different professional groups.

## 3. Results

Results are reported in alignment with the structure of the intervention and the three core domains of evaluation: medical effectiveness, economic feasibility, and work psychological and organizational adaptation. Together, these findings provide insight into the system-level impacts of digitally supported, regionally embedded emergency care models and their relevance for personalized medicine in rural settings.

### 3.1. Medical Results

The medical evaluation assessed the RuralRescue model’s capacity to enhance prehospital emergency care through earlier, context-sensitive, and data-driven interventions. Results are reported along the three fully implemented pillars of the intervention: community-based CPR training (Pillar 1), activation of professional first responders via the Land|Retter app (Pillar 2), and tele-emergency physician support (Pillar 3). Each pillar contributed in different ways to advancing the principles of personalized emergency medicine—defined here as care that is responsive to individual clinical, geographic, and organizational context.

#### 3.1.1. Pillar 1: Layperson CPR Training and Early Intervention

Over the course of the project, 12,600 citizens in the district of Vorpommern-Greifswald were trained in basic life support (BLS) using the nationally standardized “Prüfen-Rufen-Drücken” (Check–Call–Compress) model. Trainings were adapted to various community contexts, including schools, public events, and institutions, and included both in-person and digital learning components [28]. Post-training surveys indicated increased confidence and competence in handling emergency scenarios.

In out-of-hospital cardiac arrest (OHCA) cases with bystander CPR—triggered either by direct witnessing or informal proximity—the return of spontaneous circulation (ROSC) rate was 35.6 percent, exceeding the rural German average of 27 to 30 percent [26]. This demonstrates that early, community-based intervention significantly improves survival outcomes and exemplifies temporal and social individualization, enabling localized and non-professional response when formal care access is delayed.

#### 3.1.2. Pillar 2: First Responder Activation via the Land|Retter App

During the implementation period, 9233 alerts were transmitted via the Land|Retter mobile application to a pool of professionally qualified responders, including off-duty emergency physicians, nurses, and paramedics. Each participant—referred to as a “Rural Saver” (LandRetter)—underwent standardized onboarding, including validation of clinical competencies and participation in an emergency readiness orientation [28].

The app’s GPS-based alerting algorithm enabled dispatch centers to identify and notify the most suitable nearby responder based on both geographic proximity and professional qualification. In 44.3 percent of activations, a LandRetter arrived on scene before the EMS unit, achieving a median lead time of 4 min and 24 s [28]. This reduction in the therapy-free interval is especially relevant in remote areas where ambulance arrival may be delayed.

The Land|Retter system exemplifies spatial and operational personalization of emergency care. By matching responder profiles to emergency needs in real time, it enabled rapid deployment of medical support tailored to the situational geography and available human capital. Usability and acceptance surveys among participants yielded satisfaction ratings exceeding 4.2 on a 5-point scale, indicating high confidence in the system’s reliability and effectiveness [28].

#### 3.1.3. Pillar 3: Tele-Emergency Physician (TEP) Support

The tele-emergency physician (TEP) system was used in 3611 EMS cases across the project period. The system was deployed in cases where physician input was clinically indicated but direct on-site dispatch was either unnecessary or operationally unfeasible. Paramedics transmitted real-time clinical data—including vital signs, structured assessments, and ECGs—to the remotely located physician using the secure TeleDoc interface [23].

In 17.8 percent of TEP-supported cases, physicians delegated pharmacological treatment, most commonly involving analgesics, antihypertensives, and anti-epileptics. These interventions were made based on patient-specific data and symptom profiles transmitted from the field, allowing for individualized pharmacologic decision-making without requiring physical physician presence [23].

**Figure 3:**
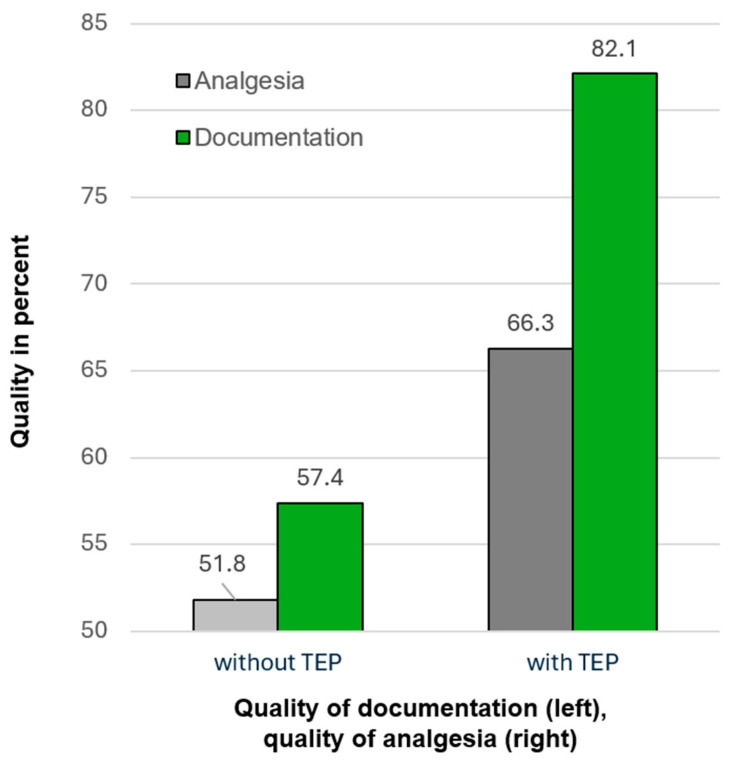
Comparison of analgesia effectiveness and documentation of hemodynamic baseline monitoring in prehospital emergency care with and without tele-emergency physician (TEP) support.

During the evaluation period, a total of 2935 patients were managed with the support of a tele-emergency physician (TEP). Of these, 362 patients (12.3%) reported a pain intensity of ≥7 on a numerical rating scale (NRS, range 0–10). Follow-up patient interviews indicated that effective pain relief was achieved in only 51.8 percent of comparable cases without TEP support, whereas the rate was significantly higher in the TEP-supported group at 66.3 percent (*p* < 0.05; see Figure 3; cf., [29]).

In addition, documentation quality was evaluated with respect to baseline hemodynamic monitoring, defined as the recording of key vital signs including blood pressure, heart rate, and peripheral oxygen saturation. This basic monitoring is essential for subsequent therapeutic decisions. It was documented in 57.4 percent of cases at the beginning of care and prior to hospital transfer in the non-TEP group, compared to 82.1 percent in TEP-supported cases (*p* < 0.001; cf., Figure 3). Importantly, 24.3 percent of all TEP-supported cases were resolved without hospital transport. In the majority of these cases (82%), the clinical presentation was deemed non-urgent and manageable by general practitioners or self-care. These case-specific triage decisions illustrate how telemedical supervision enables flexible, context-responsive routing of patients, thereby avoiding overtreatment and unnecessary resource consumption [23,30].

In terms of diagnostic accuracy, a comparison of prehospital assessments with hospital discharge data (*N* = 323) yielded an 84.9 percent diagnostic concordance rate across tracer conditions, including stroke, acute coronary syndrome, and COPD exacerbation [23]. Documentation completeness in TEP-supported cases was 92.1 percent driven by the structured data entry format of the TeleDoc system. This supports not only real-time decision-making but also retrospective quality assurance and system-level learning [30].

Together, these results show that the RuralRescue model improved clinical outcomes and optimized EMS workflows while embedding personalization at multiple levels. Through community engagement, professional responder matching, and remote physician support, care was tailored to the patient’s clinical condition, location, and context-specific care options.

### 3.2. Economic Results

The analysis showed that decentralized TEP services improved system responsiveness while reducing overall operational costs, particularly in sparsely populated regions with long deployment routes [24]. The total annual full cost of operating a decentralized TEP unit on a 24/7 basis was calculated at EUR 696,949 (≈USD 766,600). This included EUR 148,876 (≈USD 163,800) in fixed costs (office infrastructure, administration) and EUR 548,073 (≈USD 602,800) in variable and jump-fixed costs, such as staff salaries, telemedicine equipment, and licenses [31]. While the system provided clinically valuable support, its average usage—3.96 cases per day with a mean service time of 24.6 min—resulted in idle time exceeding 90 percent, indicating substantial overcapacity in single-district settings [31].

To address this, a queuing system model (M/M/k; ∞, FIFO) was used to simulate alternative coordination scenarios. The modeling showed that one TED could effectively support up to 36 ambulances while keeping the probability of delayed physician contact below 5 percent, a level considered acceptable for most prehospital situations. Under a centralized model, where one TED supports 20 districts, the cost per district drops to EUR 57,300 (≈USD 63,000)—a reduction of over 90 percent compared to standalone district operation [31].

**Figure 4:**
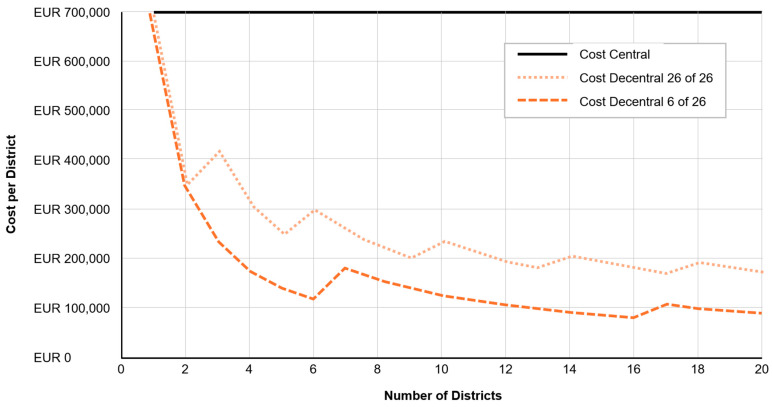
Cost functions of different scenarios of central or decentral tele-emergency services [11].

These findings confirm that personalized physician access—delivered based on real-time need, clinical data, and EMS telemetry—is not only medically feasible but also economically sustainable when scaled regionally. As Figure 4 shows, the unit cost depends strongly on the number of districts joining the tele-emergency center.

The system enables dynamic resource allocation, where expert supervision is activated for high-acuity cases and withheld in less critical scenarios, reflecting demand-sensitive personalization of care intensity. This approach supports both individualized patient management and strategic resource stewardship, aligning with core principles of value-based personalized emergency medicine.

### 3.3. Formative Work Psychological and Organizational Results

The formative evaluation assessed how different professional groups within the RuralRescue model adapted to the digital and structural innovations introduced by the project. It focused on workload perception, job satisfaction, knowledge management, technology acceptance, and regional integration—factors that critically influence the success of personalized, data-enabled emergency care models in real-world settings.

#### 3.3.1. Workload and Job Satisfaction

Workload was measured using the NASA Task Load Index (NASA-TLX) and job satisfaction via standardized Likert-based self-assessments. Results revealed differentiated patterns: Experienced EMS professionals, including long-serving paramedics and emergency physicians, reported increased job satisfaction following implementation of the new care model. In contrast, less experienced staff—especially newly trained paramedics—indicated heightened workload, transitional stress, and role uncertainty during the early phases of adoption [32,33]. The study also found that emergency teams with lower satisfaction scores reported higher degrees of informal knowledge sharing across professional boundaries [19].

Interestingly, the data also showed that higher workload levels were positively correlated with digital tool acceptance, particularly among dispatchers and field personnel. This suggests that digital supervision and documentation tools were perceived not as burdens, but as adaptive supports under pressure, reinforcing their value for role-specific adaptation [33].

**Figure 5:**
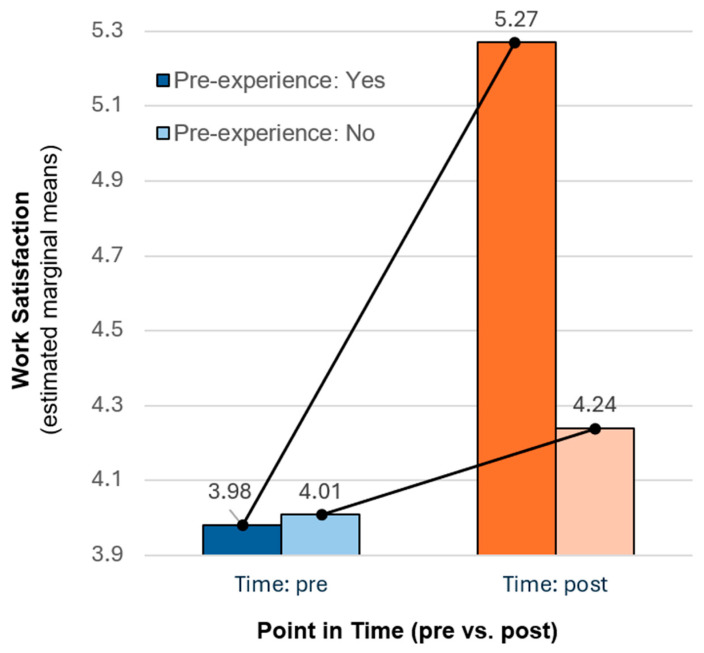
Interaction effect of time and prior telemedical experience on work satisfaction.

Work satisfaction improvements were found to depend on prior digital experience. Work satisfaction was measured using a validated polarity scale from 1 (low satisfaction) to 10 (high satisfaction) [34]. The study found a significant interaction between time (pre vs. post digital transformation) and prior experience with telemedicine. While overall satisfaction increased after the implementation of tele-emergency physicians (TEPs), this effect was statistically significant only for participants with prior telemedical experience (pre = 3.98 vs. post = 5.27, F (1315) = 4.09, *p* < 0.05). In contrast, no comparable increase was observed among less experienced staff (pre = 4.01 vs. post = 4.24; see Figure 5). These results suggest that experience in digital systems moderates satisfaction gains during technology-driven innovation in emergency care.

#### 3.3.2. Knowledge Sources and Knowledge Transfer

Knowledge management was evaluated using the KODE^®^ Competence Atlas framework, which classified sources of knowledge into four quadrants (personal/organizational × explicit/implicit). The evaluation found that informal knowledge transfer—including peer coaching, mentoring, and spontaneous collaborative problem-solving—was particularly high among staff with lower job satisfaction [31]. This indicates that interprofessional learning served as a compensatory mechanism, helping to bridge gaps in experience or protocol clarity.

The integration of telemedical feedback and digital case documentation further enabled context-sensitive learning loops, contributing to individualized professional development. These findings suggest that the system’s learning infrastructure supports individualization not only for patients, but also for clinical decision-makers.

#### 3.3.3. Technology Acceptance

Technology acceptance was measured using an adapted technical acceptance model (TAM), which included perceived usefulness, ease of use, and intention to adopt. Overall, acceptance of digital elements such as the tele-emergency physician interface and structured documentation systems was moderate to high, with notable variation across roles. Nurses and paramedics demonstrated the highest acceptance levels, followed by dispatchers; emergency physicians were more cautious, particularly regarding real-time decision transparency and medicolegal responsibility [30,31].

Notably, technology acceptance was strongly influenced by perceived task complexity and workload. Staff in high-stress roles were more likely to view digital tools as personally beneficial supports, reinforcing the idea that personalized system design should consider cognitive load and user-specific needs.

#### 3.3.4. Regional and Structural Acceptance

The GEM Assay (adapted from the Global Entrepreneurship Monitor framework) was applied to assess regional attitudes toward innovation and structural change. The results showed broad support for the RuralRescue model among both frontline professionals and institutional stakeholders. Regional identity and a shared understanding of local healthcare challenges enhanced the acceptance of digitally supported models of care [30].

Stakeholders particularly appreciated the modular structure of the intervention, which allowed individual components (e.g., first responder system, TEPs) to be integrated based on regional capacity and readiness. This structural adaptability is a prerequisite for scalable personalized systems across diverse rural settings.

#### 3.3.5. Relevance to Personalized Emergency Medicine

The formative evaluation demonstrates that personalization in emergency care extends beyond the patient to include providers, workflows, and digital interfaces. Variability in stress experience, technology acceptance, and knowledge behavior across professional roles underscores the need for context-aware implementation strategies. Tailoring support structures, training intensity, and digital onboarding to match professional background and regional infrastructure is essential for sustaining personalization at the system level. These findings align with the broader definition of personalization, encompassing not only clinical decision-making but also the social, psychological, and organizational factors that enable individualized care delivery in complex environments [29,30].

## 4. Discussion

The RuralRescue project demonstrated that personalized emergency care can be effectively implemented in rural regions through the coordinated use of digital technologies, targeted training, and adaptive care structures. The evaluation revealed improvements in clinical performance, resource efficiency, and organizational integration across all three implemented pillars of the model.

### 4.1. Summary of Main Findings

From a medical perspective, community-based CPR training (Pillar 1) significantly enhanced early intervention capacity, contributing to a 35.6% ROSC rate in OHCA cases—above the national rural average. The app-based responder activation system (Pillar 2) enabled geo-personalized mobilization of trained professionals, shortening the therapy-free interval by a median of over four minutes in nearly half of the activations. The TEP system (Pillar 3) supported remote diagnostics and delegated therapy in 3611 cases, with 17.8% involving physician-authorized medication and 24.3% avoiding unnecessary hospital transport. Diagnostic concordance with hospital data reached 84.9%, and digital documentation completeness exceeded 92%, demonstrating clinical precision and accountability.

Economically, decentralized TEP units proved effective but cost-intensive at the district level. Simulation models showed that centralized coordination could cut per-district costs by over 90%—from EUR 696,949 to EUR 57,300—without sacrificing service quality, assuming optimized TED-to-ambulance ratios. These results support the scalability of demand-sensitive personalization.

The organizational evaluation revealed divergent responses to digital transformation: experienced personnel reported greater job satisfaction, while less experienced staff indicated higher stress. Technology acceptance correlated positively with perceived workload and varied by role, with paramedics and nurses more receptive than physicians and dispatchers. Informal knowledge sharing emerged as a compensatory strategy, and stakeholder surveys confirmed broad regional support for the model’s structural innovations.

Taken together, the findings demonstrate that personalized emergency care is feasible in rural areas when tailored to patient needs, regional infrastructure, professional diversity, and systemic constraints. RuralRescue offers a multi-dimensional proof of concept for integrating real-time clinical data, digital tools, and local capacity to enable individualized, value-based care in underserved regions.

### 4.2. Contributions to Personalized Emergency Medicine

The RuralRescue project demonstrates how personalized medicine can be operationalized in emergency care through the integration of digital tools, professional competencies, and real-time clinical data. Although not initially framed under the label of personalized medicine, the model supports key personalization mechanisms across the care continuum—from early intervention to triage, therapy, and system learning.

The tele-emergency physician (TEP) system exemplifies real-time clinical personalization. In over 3600 cases, remote physicians made diagnostic and therapeutic decisions using live ECGs, vital signs, structured assessments, and visual input. Nearly 18% involved delegated medication, tailored to both patient-specific data and the competencies of the on-site team. This situational, data-driven delegation aligned care intensity with clinical need rather than provider availability, enhancing precision in resource-limited rural areas.

TEP support also enabled context-aware triage: 24.3% of cases were managed without hospital transport, reflecting personalized decisions that minimized overtreatment, transport risks, and systemic strain.

The Land|Retter responder system implemented geo-personalized early intervention via a mobile app that alerted trained responders based on location and qualification. This real-time localization extended emergency coverage, adapting to local infrastructure, community layout, and responder distribution.

At the system level, over 250,000 EMS data points were collected, enabling longitudinal monitoring of outcomes, triage, and documentation. These data form the basis for adaptive learning and protocol refinement aligned with real-world trajectories.

Personalization was also advanced through role-specific training and workflow integration. Paramedics, dispatchers, and physicians received differentiated onboarding and developed collaborative competencies essential for tele-supervised care, supporting individualized professional development and smoother digital integration.

Formative findings highlight that implementation success depends on psychological and organizational factors. Technology acceptance varied by role, workload, and prior experience, underscoring the need to adapt tools and training to users’ identities and stress profiles.

In sum, RuralRescue illustrates that personalized emergency medicine extends beyond molecular diagnostics to include dynamic alignment of care with patient status, professional readiness, regional resources, and digital infrastructure—a replicable framework for systems-level personalization in underserved regions.

### 4.3. Limitations

This article presents a secondary analysis of the RuralRescue project through the lens of personalized emergency medicine. The project was not originally designed to evaluate personalization but aimed at improving emergency care access and quality in a rural setting. Consequently, this re-analysis must be interpreted with caution.

The evaluation employed a pre–post design without a control group, consistent with common practice in implementation research under real-world constraints [25]. While this design ensured ecological validity, it limits causal attribution and increases vulnerability to confounding influences such as concurrent system changes, demographic shifts, or policy adjustments. Selection bias may have occurred due to non-random allocation of intervention components, with access to tele-emergency physician (TEP) support, responder alerts, or training programs influenced by dispatcher discretion, digital readiness, and geographic factors. Self-selection effects may also have influenced survey participation. Observer bias is a further limitation, particularly in self-reported outcomes related to work satisfaction, stress, and acceptance. These subjective measures are inherently prone to social desirability effects and role-dependent interpretation.

Confounding variables such as prior professional experience, local EMS infrastructure, and team composition may have moderated individual responses and organizational outcomes but were not fully controlled. Moreover, not all outcome indicators were available for the entire sample. Clinical metrics such as diagnostic concordance or triage decisions were analyzed only for subsets of cases, limiting generalizability.

Finally, integration with primary care—initially planned as a fourth intervention pillar—was not realized. As a result, the evaluation cannot address care continuity beyond the acute emergency phase or assess intersectoral personalization strategies. Despite these limitations, the RuralRescue project provides a robust, real-world foundation for understanding how personalization mechanisms can emerge from integrated emergency interventions in rural systems.

### 4.4. Recommendations for Further Research

While RuralRescue demonstrated the feasibility of personalized, digitally supported emergency care in rural regions, several areas merit further investigation to validate and extend these findings. Future research should apply controlled comparative designs, such as randomized or matched cohort studies, to strengthen causal claims. This is particularly relevant for assessing the impact of tele-delegated medication, triage accuracy, and diagnostic concordance under TEP supervision.

The extensive dataset generated by the project offers a foundation for predictive modeling and AI-based decision support. Tools such as adaptive triage algorithms and responder routing optimization could further personalize care and improve system responsiveness in real time. Studies should explore intersectoral integration, linking EMSs with out-of-hours care and hospital services to ensure continuity across the patient journey. While not realized in RuralRescue, this remains a critical area for delivering fully personalized, longitudinal emergency care.

Further research into the legal, ethical, and professional governance of remote clinical authority is essential. As tele-delegation increases, clear frameworks are needed to ensure safety, accountability, and acceptance by healthcare providers. Moreover, the variation in technology acceptance across roles suggests the need for personalized implementation strategies. Tailoring onboarding, digital training, and workflow redesign to staff roles, stress profiles, and institutional culture may enhance long-term adoption.

Lastly, disaggregated analysis of RuralRescue data could identify how personalization functions for underserved groups such as elderly patients or residents of isolated areas, helping refine equity-oriented design in rural EMS systems. These directions emphasize the need for interdisciplinary research that integrates clinical, technological, ethical, and organizational dimensions to advance system-level personalization in emergency medicine.

### 4.5. Practical Implications

The RuralRescue model demonstrates that personalized emergency care can be operationalized through digital infrastructure, tailored training, and adaptive policy—impacting clinical practice, organizational structures, and regulatory frameworks.

#### 4.5.1. Individual and Clinical Practice Level

Customized digital workflows supported real-time TEP decision-making, enabling individualized diagnostics and therapy based on live clinical data. Paramedics delivered situation-specific care under remote oversight, particularly beneficial in high-acuity and resource-limited settings. The system’s support for data-driven triage—24% of TEP-supervised cases managed without transport—aligns with value-based, risk-adjusted care models.

The Land|Retter app enabled geo-personalized responder mobilization based on proximity and verified competencies, improving early intervention in underserved areas. Role-specific training fostered telemedical integration and interprofessional collaboration, underscoring the role of personalized education in EMS transformation.

#### 4.5.2. Organizational and System Level

RuralRescue’s digital case documentation (over 250,000 records) provided a basis for longitudinal analysis, protocol refinement, and quality improvement. Technology acceptance varied by role, workload, and job satisfaction, highlighting the importance of context-aware implementation strategies tailored to human and organizational factors. Centralizing TEP coordination—as shown in simulation models—significantly reduced per-district costs while maintaining responsiveness, supporting economically sustainable personalization.

#### 4.5.3. Policy, Legal, and Regulatory Implications

The project underscores the need for updated legal frameworks enabling remote medical delegation, digital consent, and real-time data protection. As a result, the state of Mecklenburg-Western Pomerania initiated the creation of a centralized tele-emergency center, following the project’s economic recommendations. Long-term integration of TEP services with statutory on-call physicians and primary care could facilitate cross-sector continuity, advancing personalized emergency care as part of a cohesive, patient-centered system.

## 5. Conclusions

The RuralRescue project demonstrates that personalized emergency care is both feasible and effective in rural contexts when supported by digitally integrated, role-specific, and data-driven systems. Through real-time telemedical supervision, geo-personalized responder deployment, and adaptive workflows, the model advances core principles of personalized medicine—tailoring interventions not only to clinical presentation but also to geographic, organizational, and human resource factors. These findings offer a transferable framework for implementing individualized, value-based emergency services in structurally underserved regions and can be applied to other rural areas of hard-to-access environments.

## Data Availability

The full results including statistical data output, all surveys and tools as well the final report of the project, are open to the public at https://innovationsfonds.g-ba.de/projekte/neue-versorgungsformen/landrettung.63 (accessed on 7 February 2025).

## Abbreviations

The following abbreviations are used in this manuscript:

CPR	Cardiopulmonary Resuscitation
TEP	Tele-Emergency Physician
EMSs	Emergency Medical Services
ROSC	Return of Spontaneous Circulation
OHCA	Out-of-Hospital Cardiac Arrest
G-BA	Gemeinsamer Bundesausschuss (Federal Joint Committee)
AED	Automated External Defibrillator
FIFO	First-In-First-Out
M/M/k, ∞, FIFO	M/M/k Infinity FIFO (Economical Queuing Model)
